# Cardiac eccentric remodeling in patients with rheumatoid arthritis

**DOI:** 10.1038/s41598-018-24323-0

**Published:** 2018-04-12

**Authors:** Valeria Pascale, Rosa Finelli, Rocco Giannotti, Enrico Coscioni, Raffaele Izzo, Francesco Rozza, Dario Caputo, Paolo Moscato, Guido Iaccarino, Michele Ciccarelli

**Affiliations:** 1 Department of Medicine, Surgery and Dentistry, University of Salerno, Salerno, Italy; 2 Department “Cuore”, University Hospital “San Giovanni di Dio e Ruggi d’Aragona”, Salerno, Italy; 3 Department of Advanced Biomedical Sciences, Federico II University of Naples, Salerno, Italy; 4 Department of Medicine, University Hospital “San Giovanni di Dio e Ruggi d’Aragona”, Salerno, Italy

## Abstract

It is known that patients with rheumatoid arthritis (RA) have a higher risk of coronary heart disease and sudden cardiac death. Abnormalities in cardiac geometry appear to be involved in the setting of the cardiovascular risk, but it has never been specifically investigated in RA. We enrolled 44 patients with RA compared to 131 subjects without RA (normal, N): The RA aged between 18 and 70 years (mean 48.3 ± 2.1), 25 females, BMI 27.6 ± 0.9; N, of equal age (48.6 ± 1.2, n.s.), included 80 females (BMI 26.7 ± 0.2, ns). Cardiac Ultrasounds showed an increase of the diameter of the left ventricle but not in the septum with reduction of relative wall thickness (RWT) in the RA population compared to N. Relative wall thickness inversely correlates with biochemical parameters of inflammatory response (gamma globulin, p < 0.03; F = 5,660) and anti citrullinated peptides antibody (anti-CCP Ab) (p < 0.02; F = 7,1620) We conclude that unfavorable cardiac remodeling can increase cardiovascular risk in patients with RA.

## Introduction

Rheumatoid arthritis (RA) is a chronic inflammatory disease with an autoimmune pathogenesis with a very high risk of invalidity (80% of cases) and reduced survival^1,2^. The incidence rate of RA differs between 20 and 50 new cases every 100,000 people per year in North American and European countries and prevalence of the disease is higher in women^3–6^. RA affects mainly the diarthrosis joints, especially the proximal interphalangeal, metacarpophalangea, wrists, metatarsophalangeal, knees, elbow, ankle, scapular-humeral, hip and cervical spine, temporo-mandibular joint. RA has a multifactorial genesis, where genetic, environmental and random factors contribute to the development of the disease^7^. Among these latter, smoking and irritants are prevalent in patients that are positive to rheumatoid factor (RF) and/or to the anti-citrullinated peptides (anti-CCP) antibody. Several epidemiological studies have observed the association between the systemic autoimmune disorders and cardiovascular disease with this last as the prevalent cause of death in patients with RA^8^. This association, albeit well described, remains largely unexplained. Indeed, cardiovascular diseases are the main cause of death and disability in western countries, prevalently due to atherosclerotic lesions of the vascular tree^9^. Cardiovascular complications can be predicted in the general population on the basis of the number risk factors as collectively considered, which all together concur to the definition of the “global cardiovascular risk”, and include parental history, lifestyle and unhealthy habits. The recently developed concept of the cardiovascular continuum^10^ has also introduced the research of subclinical organ damage for a better definition of the cardiovascular risk. In particular, changes in cardiac geometry including eccentric or concentric remodeling, associated with increase in left ventricle size can be assessed easily by cardiac ultrasounds (CUS), and allow to detect those modification that are more often associated with the risk of cardiovascular events^11–13^. Several findings have shown the presence of diastolic dysfunction without manifest cardiac disease^14–17^ in patients with RA as compared to control patients not affected by either RA or cardiovascular disease. Therefore, the employment of early markers of cardiac disease can possibly help to identify RA patients at higher risk to develop cardiac failure. In this study, we investigated the role of CUS-assessed eccentric remodeling in setting up the cardiovascular risk of patients with RA.

## Materials and Methods

### Population

For this study we enrolled 44 patients with diagnosis of RA according to the ACR/EULAR 2010 criteria^18^, from the Rheumatology Division of AOU San Giovanni di Dio and Ruggi d’Aragona. Patients with known cardiovascular conditions, including hypertension, cardiac ischemia, heart failure, diabetes, valves diseases, pulmonary and aortic complications were excluded. As control group, we selected 131 subjects, not suffering of RA or cardiovascular disease from our outpatient clinic of the San Giovanni di Dio e Ruggi d’Aragona Hospital. All parameters, including anamnesis, physical examination, biochemistry, EKG, cardiac and vascular ultrasound and ambulatory blood pressure monitoring were collected during ambulatory visits and stored in the server of the Hospital. All procedures were performed in accordance with the EULAR Guidelines. Written informed consent was obtained from patients. The protocol was approved by the Ethical Committee of the San Giovanni di Dio e Ruggi d’Aragona Hospital, Salerno.

### Clinical and biochemical assessments

Disease activity according to the DAS28 and the degree of disability by HAQ were evaluated^19^. A blood sample was collected after 10 h fasting to measure RF, anti-CCP, antinuclear anti-body (ANA), C-reactive protein (PCR), Erythrocyte sedimentation rate (ESR). Moreover, all patients were subjected to mono-dimensional (M-mode), bi-dimensional (B-mode), Doppler and color Doppler cardiac ultrasound (CUS) and via CUS probe 5-1 MHz. We evaluated: interventricular septum (IVSTd) and posterior wall thickness in diastole (LVPWTd), ventricular end-diastolic and end-systolic diameters (LVIDd, LVIDs) aortic root diameter, the end-diastolic diameter of the atrium. Relative Wall Tickness (RWT) was calculated according to the formula: (IVSTd + LVPWTd)/ LVIDd.

### Statistical analysis

A Power Analysis was performed to identify the sample size required to detect a difference in cardiac size of at least 7 mm with power of 85%. Anthropometric, biochemical and cardiac ultrasound parameters are expressed as mean ± standard error. Differences among patients and controls were analyzed by unpaired Student’s t test; relationships between variables were considered by linear correlation. All data were analyzed using Prism 6.

## Results

### Biochemical and CUS parameters

We considered 44 RA patients (group 1), aged between 18 and 70 years, including 25 females, and a control group (group 2) consisting of 131 healthy subjects (80 females) not affected by RA and CVD. The anthropometric parameters of the two groups were similar (Table 1). Similarly, the two groups did not show differences in the metabolic profile and renal function (Table 2). Comparing echocardiographic parameters between the two populations, we observed a statistically significant increase in both LVIDd and LVIDs in patients with RA, without differences in IVSTd and LVPWTd (Table 3). Eccentric dilatation is also confirmed by RWT, which is significantly decreased in patients with RA as compared to control, indicating a prevalent left ventricle eccentric remodeling in the RA group (Table 3).

**Table 1:** RA patients (group 1) and control subjects (group 2) are similar for anthropometric parameters. SBP: systolic blood pressure; DBP: diastolic blood pressure; BMI: body mass index.

	Age (year)	Woman %	Man %	SBP (mmHg)	DBP (mmHg)	Weight (Kg)	Height (cm)	BMI (Kg/m^2^)
Group 1	48,33 ± 2	57%	43%	123,6 ± 2	75,8 ± 1	73,3 ± 2,7	162,5 ± 1,6	27,66 ± 1
Group 2	48,56 ± 1	61%	39%	125 ± 1	74,2 ± 0,73	70,2 ± 0,73	162,62 ± 1	26,56 ± 0,2
P value	n-s			n-s	n-s	n-s	n-s	n-s

**Table 2:** RA patients (group1) and control subjects (group2) are similar for biochemical parameter. TC: total cholesterol, c-LDL: cholesterol- LDL, c-HDL: cholesterol- HDL, TG triglyceride, CKD-EPI equations for glomerular filtration rate.

	Glycaemia mg/dl	TC mg/dl	c-LDL mg/dl	c-HDL mg/dl	TG mg/dl	CKD-EPI ml/min
Group 1	91 ± 7	198,3 ± 5	113 ± 5	67,14 ± 3,3	91 ± 4	100,9 ± 22,3
Group 2	94,4 ± 3,2	215 ± 6,3	147 ± 6,2	50,7 ± 2,5	123 ± 13,1	105,4 ± 18,6
P value	n.s.	n.s.	n.s.	n.s.	n.s.	n.s.

**Table 3:** Patients with RA (group 1) have left ventricle systolic and diastolic diameter greater than controls (group 2). Similarly, left atrium has a major diameter in patients of group 1. RWT is lower in patients with RA than control group.

	LVIDs mm	LVIDd mm	IVSTd mm	LVPWTd mm	Ao mm	LA mm	RWT
Group 1	33 ± 1	56,8 ± 0,2	10,1 ± 0,2	7,78 ± 0,2	31,1 ± 1	33,7 ± 0,7	0,32 ± 0,01
Group 2	28,9 ± 0,2	47,5 ± 0,2	9,7 ± 0,2	8,85 ± 0,1	32,03 ± 0,2	35,15 ± 0,2	0,39 ± 0,01
P value	<0,01	<0,01	0,1 (n.s.)	0,2 (n.s.)	<0,05	<0,01	<0,01

### Association of RWT and biochemical patterns in the population affected by AR

**Figure 1:**
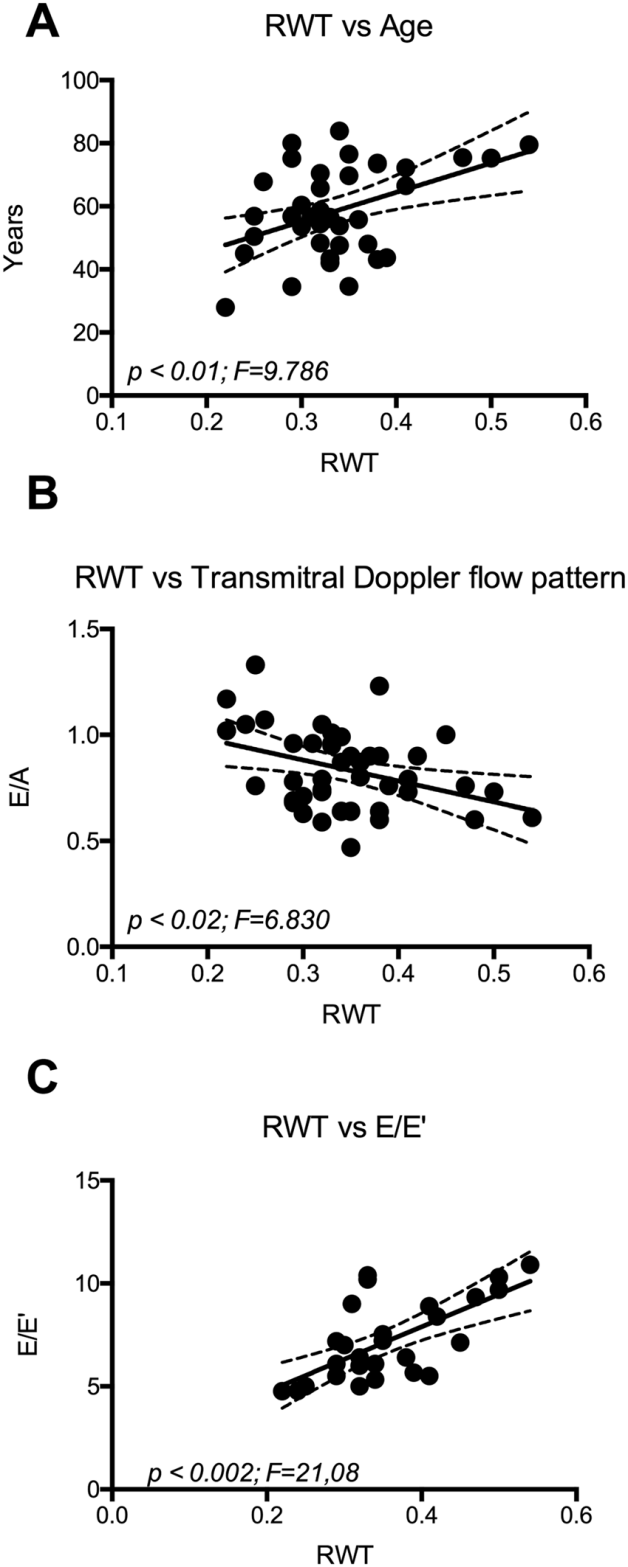
Cardiac concentric remodeling, estimated by RWT, is associated to diastolic dysfunction (Panel A,B) and directly correlates to age (Panel C).

**Figure 2:**
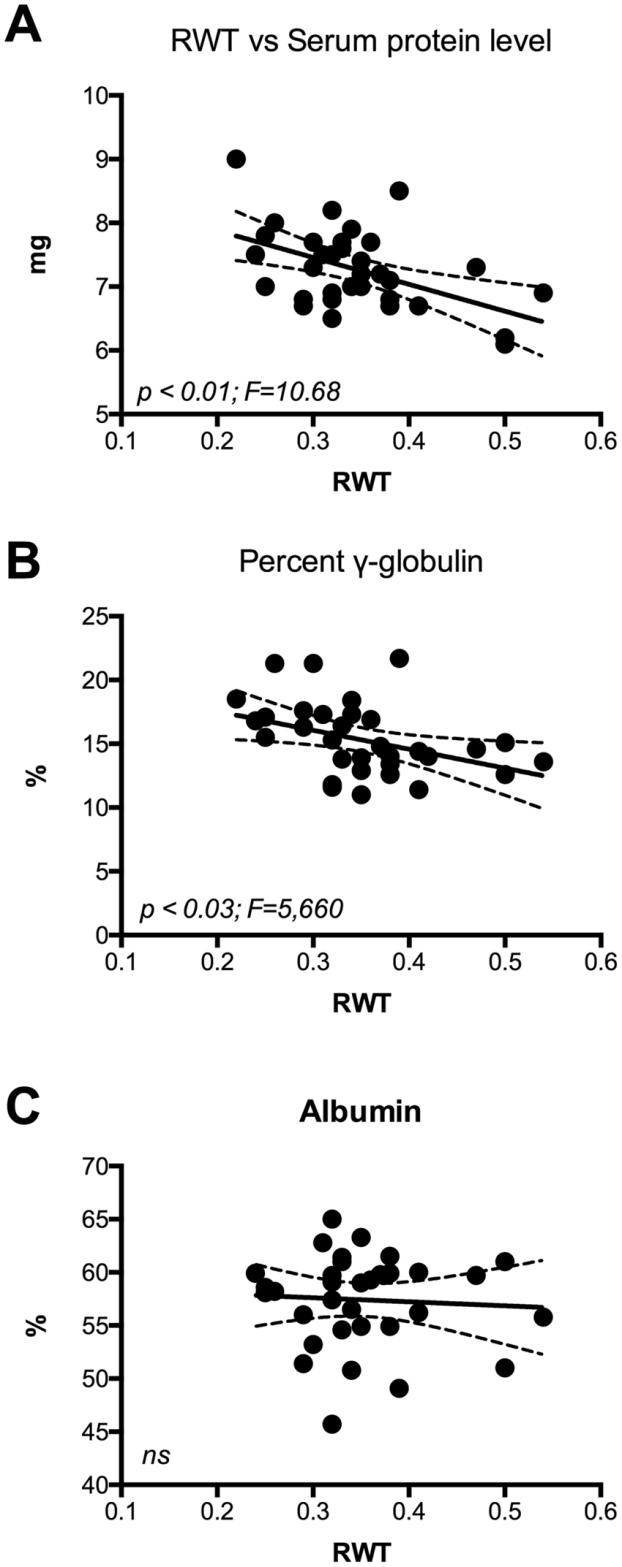
RWT inversely correlates with the dose of the total protein (Panel A) and the level of gamma globulin (Panel B); no correlation was found between RWT and serum albumin (Panel C).

**Figure 3:**
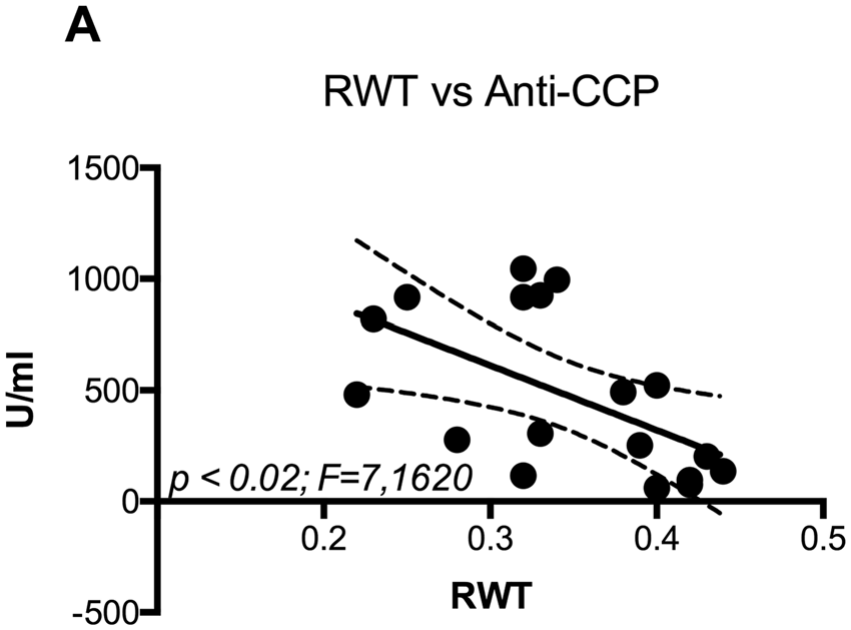
RWT inversely correlates to level of anti-peptides citrullinated (anti-CCP) antibody. In RA patients with anti-CCP Ab values between 60 and 2000 U/ml and aged between 40 and 60 years old we observed that RWT decreases in patients with higher level of anti-CCP Ab.

Patients with eccentric remodeling are considered at higher CVR. In order to identify possible determinants of eccentric remodeling in patients with RA, we evaluated through correlation analysis the impact of biochemical variables on the left ventricle geometry. We observed that in patients with RA, RWT directly correlates to age (Fig. 1A) and inversely correlates to the diastolic function as evidenced by E/A ratio (Fig. 1B) and E/E’ ratio (Fig. 1C). These findings are consistent with previous literature^20^ and validate the observation in our study group. In RA group, RWT value inversely correlates with the levels of total plasma proteins (Fig. 2A) and gamma globulin (Fig. 2B), but no significant correlation was found with the serum albumin level (Fig. 2C). Anti-CCPs are specific markers of RA and they can be found at the early stage of disease^21,22^ and serum levels > 60 U/ml indicate advanced RA condition. In our population, the highest level of anti-CCP was found in patients between fifth and sixth decade of age. Therefore, we selected patients with RA with anti-CCP Ab values between 60 and 2000 U/ml and aged between 40 and 60 years old. In this subgroup, we observed a significant inverse correlation between RWT and anti-CCP level (Fig. 3).

## Discussion

Our study shows for the first time that RA patients present cardiac eccentric remodeling before clinical features of cardiovascular disease and in absence of the common cardiovascular risk factors. Our observations confirm and extend previous findings of reduced diastolic function in RA^14^ and are in line with recent publication evaluating alterations of cardiac geometry in RA patients. In particular, Fine *et al*.^23^ studied the myocardial deformation during ventricular contraction and relaxation by using speckle tracking echocardiography (STE). There, it was revealed that the longitudinal structural of the left and right ventricular wall at systolic peak is significantly worsened in patients with RA with preserved diastolic function. The result persisted after adjustment for age, gender, blood pressure, BMI and heart rate and also after comparing patients with controls. These data support the idea that measuring shortening abnormalities is a more sensitive parameter than diastolic dysfunction to detect presence of cardiomyopathy in subclinical phase, at least in RA population. Our data confirm that an early sign of cardiac damage is a feature of RA, and add the consideration that RWT being a parameter of easy consultation, internists can measure it with a standard CUS, and therefore assess it in all patients affected by RA, while STE being still an instrument available only to expert CUS laboratories is mainly available to cardiologists.

Our data show that aging directly correlates with RWT in RA patients as well as in controls. This is somewhat expected^20^, but this phenomenon might attenuate our finding of reduced RWT in the aged population (i.e. >65 years old). When limiting the analysis of RWT to patients <65 yrs old, though, we noticed that patients with the longest history of disease (>15 yrs) showed a tendency towards the lowest RWT (data not shown). This latter finding is consistent with previous literature^14^. Indeed, in the Lugo study, disease patients with diastolic dysfunction were older than those without this abnormality^14^.

Considering that cardiac eccentric remodeling in RA cannot be attributed to clinical cardiovascular risk factors, the inflammatory hypothesis responsible for structural changes holds the most likely. The observation that RWT inversely correlates with gamma globulin, and in particular with Anti-CCP levels fits properly in this pathophysiological context. CHF can evolve from a variety of pathogenic conditions including RA. However, it still remains difficult to discern the specific weight of RA respect to the common risk factors for CHF^24^. The problem of defining the incidence of CHF in patients with RA may reside in the diagnostic process; indeed, the use of only clinical criteria for the diagnosis can result inaccurate, with both over- or lack of diagnosis. This is the case of edema of the ankles that can be confused with swollen joints and/or lung congestion interpreted as pulmonary involvement of RA. For this reason, guidelines for RA state the need to include CUS into to the diagnostic flowchart for CHF, even when is only suspected^25^. So far only CUS markers of diastolic dysfunction^26–28^ have been associated to RA^29,30^, but here we demonstrated that RWT could be added to the list since it is strictly associated to level of inflammation. This last observation can have relevant implication in the therapeutic approach. Among patients without history of cardiac ischemic disease, those with higher serum levels of IL-6, CRP, TNF appeared to have a two or four times higher risk of developing CHF compared to patients with lower levels of cytokines^31–35^. Recently, the result of the CANTOS clinical trial testing the use of Canakinumab monoclonal anti-IL1b antibody in gout patients has shown a reduction in cardiovascular events, therefore providing further support to the link between excessive inflammatory response and increased CVR^36^. Cardiac remodeling too is driven by cytokines and serum levels TNFα have been demonstrated to regulate expression of metallo proteinases (MMPs) and of their inhibitors (TIMPs) producing increase in the ratio MMPs/TIMPs, degradation of interstitial collagen fibers and development of ventricular dilatation and CHF. Along the time, however, there is an increase of the production of TIMPs responsible for a reduction in the ratio MMPs/TIMPs, from which derives an increase in the production of collagen and subsequent fibrosis and ventricular dilatation^37^. This time-dependent effect of TNF in the induction of cardiac remodeling suggests presence of an early window where interstitial fibrosis can be prevented^38^. This explains why treatment with anti TNF is effective to reverse the process of ventricular dilatation only when administered in the early stage, and the apparent contradiction of the worsening of the clinical condition induced by biological therapy with anti-TNF in patients with advanced CHF^39^. These considerations support research of early markers of cardiac remodeling in RA, and here, we, once again, have identified that RWT modifications is significantly correlated with the level of inflammatory activity of RA measured by Anti-CCP level.

Our study is relevant also in the Public Health perspective. Indeed, the quest towards sustainability of increased aging in western societies is calling for strategies of prevention, early disease detection and more efficient therapy^40–42^. In particular, chronic diseases represent an important challenge to tackle, for their global burden in terms of disability^43^. Among these, RA brings in a major burden for polytherapy, comorbidity, hospital accesses and increased disability^44^. In particular, the association of increased risk of cardiovascular events in RA is a major problem with still unanswered questions^45^. In this context, the identification of early marker of cardiovascular complications may allow early diagnosis and therapy. Previous study has indeed identified presence of diastolic dysfunction in RA population without clinical evidence of cardiovascular disease, firstly advancing the idea that prevention of cardiovascular complications in RA can be favored by early assessment of RA patients by CUS^14^. We provide a reliable, easily assessed parameter of CUS that can be performed without the assistance of specialized CUS laboratory.

In conclusion, our study suggests that the diagnostic path of RA patients should be modified flanking the rheumatology visit with a cardiologic examination as soon as possible after the first contact. Moreover, it is necessary to include CUS in the diagnostic path and risk assessment, given the evidence that cardiovascular risk for patients with RA is independent and it can be easily detected with the evaluation of RWT.

### Significance

1. Cardiac eccentric remodeling is significantly correlated with the level of inflammatory activity of RA.
2. RWT is an easy and accessible method to predict progression towards HF in all RA patients.
3. Early clinical and echocardiographic evaluation of patients with RA can prevent development of HF.
